# Phosphorylation of Suppressor of Hairless impedes its DNA-binding activity

**DOI:** 10.1038/s41598-017-11952-0

**Published:** 2017-09-19

**Authors:** Anja C. Nagel, Jasmin S. Auer, Adriana Schulz, Jens Pfannstiel, Zhenyu Yuan, Courtney E. Collins, Rhett A. Kovall, Anette Preiss

**Affiliations:** 1 Institut für Genetik (240), University of Hohenheim, Garbenstr. 30, 70599 Stuttgart, Germany; 2 Core Facility Hohenheim, Mass Spectrometry Unit University of Hohenheim, 70599 Stuttgart, Germany; 3 Department of Molecular Genetics, Biochemistry and Microbiology, University of Cincinnati College of Medicine, Cincinnati, Ohio, United States of America

## Abstract

Notch signalling activity governs cellular differentiation in higher metazoa, where Notch signals are transduced by the transcription factor CSL, called Suppressor of Hairless [Su(H)] in *Drosophila*. Su(H) operates as molecular switch on Notch target genes: within activator complexes, including intracellular Notch, or within repressor complexes, including the antagonist Hairless. Mass spectrometry identified phosphorylation on Serine 269 in Su(H), potentially serving as a point of cross-regulation by other signalling pathways. To address the biological significance, we generated phospho-deficient [Su(H)^S269A^] and phospho-mimetic [Su(H)^S269D^] variants: the latter displayed reduced transcriptional activity despite unaltered protein interactions with co-activators and -repressors. Based on the Su(H) structure, Ser269 phosphorylation may interfere with DNA-binding, which we confirmed by electro-mobility shift assay and isothermal titration calorimetry. Overexpression of Su(H)^S269D^ during fly development demonstrated reduced transcriptional regulatory activity, similar to the previously reported DNA-binding defective mutant Su(H)^R266H^. As both are able to bind Hairless and Notch proteins, Su(H)^S269D^ and Su(H)^R266H^ provoked dominant negative effects upon overexpression. Our data imply that Ser269 phosphorylation impacts Notch signalling activity by inhibiting DNA-binding of Su(H), potentially affecting both activation and repression. Ser269 is highly conserved in vertebrate CSL homologues, opening the possibility of a general and novel mechanism of modulating Notch signalling activity.

## Introduction

Metazoan development depends on a small number of fundamental, conserved signalling pathways that are employed repeatedly to govern cellular differentiation. Not surprisingly, their dysregulation is a major cause of congenital diseases, underlying the pathophysiology of a multitude of different cancers, including solid tumours and leukemias. Together, these fundamental pathways coordinate developmental processes by building up complex signalling networks with extensive molecular cross-talk^1–3^. One such pathway is the Notch signalling pathway, which takes place directly between neighbouring cells, steering them into alternate fates. Appropriate cellular differentiation, therefore, strictly depends on Notch signalling in many instances, explaining its impact on human health (for review^4–6^). The serious consequences of aberrant Notch signalling have triggered large-scale efforts aimed at understanding the regulatory mechanisms underlying Notch activity. One hallmark of these studies is that regulatory mechanisms can occur at multiple steps of signal transduction, ranging from the selection and variation of ligands, crosstalk with other signalling cascades, and various context and tissue specific modification of Notch signalling components^5–9^. Such investigative studies, however, are complicated by genetic redundancy in vertebrates, *e*.*g*. mammals have four Notch receptors and five ligands, highlighting the importance of using model organisms like *Drosophila melanogaster* to elucidate novel mechanisms of regulation.

The principles of Notch signal transduction are rather simple: the binding of ligands presented on the surface of the signalling cell triggers the cleavage of the Notch receptor in the adjacent signal receiving cell, releasing the intracellular domain NICD (Notch Intracellular Domain). NICD now becomes a transcriptional co-activator: together with co-activators of the Mastermind family (Mam) it assembles a ternary activator complex with the transcription factor CSL (CBF-1/Suppressor of Hairless/Lag-1) on Notch target genes (for review^9–11^). CSL acts as a molecular switch on Notch target genes as it can either assemble activator or repressor complexes depending on its binding partners^10^. The structure of these complexes has been analysed in several organisms: CSL contains three domains, an N-terminal domain (NTD), a beta-trefoil domain (BTD), and a C-terminal domain (CTD). DNA binding is mediated by NTD and BTD, whereas BTD and CTD are involved in the formation of the activator complex by binding to NICD and Mam, respectively^11^. In vertebrates, several co-repressors compete with NICD for the binding of BTD^10^. In *Drosophila*, repressor complex formation relies on the general Notch antagonist Hairless. Hairless binds with high affinity to the CTD of Su(H), thereby precluding the binding of NICD^12,13^. In addition, Hairless recruits the two general co-repressors Groucho and C-terminal binding protein to silence Notch target genes^14^.

As cells are under the influence of several developmental pathways, molecular cross-talk is required for their coordination^2,15^. Indeed, NICD phosphorylation affects complex formation and stability, which eventually results in either stimulation or inhibition of Notch signalling, depending on the mediating kinase and the targeted region of NICD (for review^16,17^). As several of these kinases are also an integral part of other signalling pathways they have the potential to act as the mediators of crosstalk. A prime example is the crosstalk between the epidermal growth factor receptor (EGFR) and Notch pathways in *Drosophila*. These two signalling pathways have a complex relationship, either acting in a cooperative or antagonistic manner, depending on the developmental context^8,18^. Pivotal to EGFR signalling is the Mitogen-activated protein kinase (MAPK), and two well established substrates of MAPK in *Drosophila* are the Notch signalling components Groucho (Gro) and Suppressor of Hairless [Su(H)], which are both restricted in their activity by MAPK dependent phosphorylation^15,19^. Nonetheless, there are likely to be other Notch signalling components that are modified in response to other signalling pathways.

Here, we report the identification of an additional phosphorylation site in Su(H) by a mass spectrometry approach. The identified phospho-serine 269 is located in the beta-trefoil domain (BTD) of Su(H), leaving the possibility of influencing the association of Su(H) with NICD and/or with DNA. Using phospho-site specific mutants we show that the phospho-mimetic Su(H)^S269D^ is impaired in transcriptional regulation. We find that protein complexes with NICD and Hairless form normally; however, we find that DNA binding is affected in Su(H)^S269D^. Moreover, overexpression analyses during fly development provide evidence for a restricted ability of Su(H)^S269D^ to activate and repress Notch target genes, revealing dominant negative effects at the same time. In contrast, the phospho-deficient mutant Su(H)^S269A^ behaves similarly to the wild type protein. As Ser269 is highly conserved, we propose a new mode of Notch signal regulation at the level of affecting DNA binding by the transcription factor CSL.

## Results

### Su(H) protein is phosphorylated on Serine 269 in S2 cell culture

**Figure 1:**
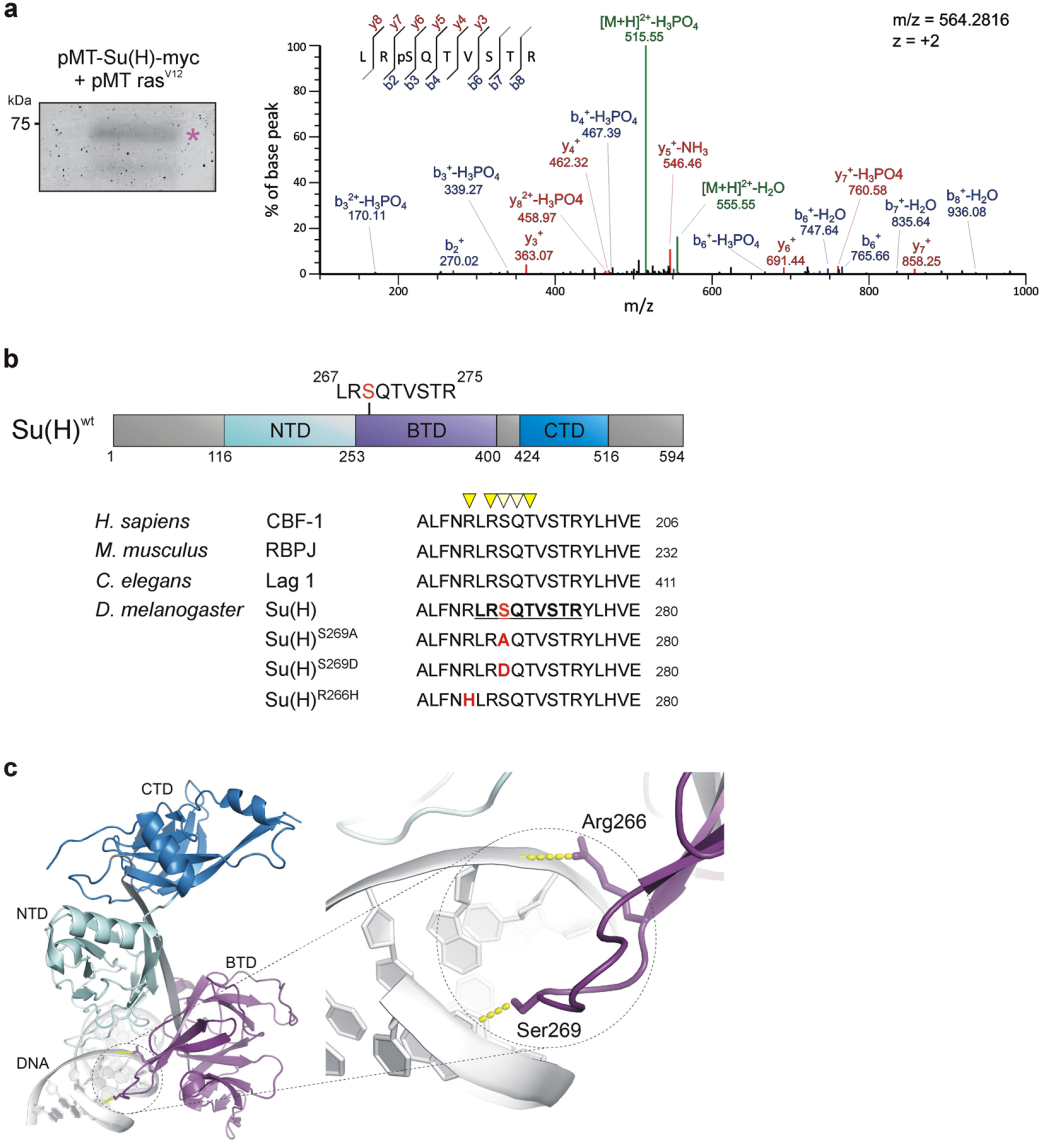
Phosphorylation of Su(H) at Serine 269. (**a**) Coomassie stained Su(H)myc protein precipitated from S2 cell culture used for mass spectrometry analyses (left, asterisk). Approximate molecular weight is given in kilo Dalton. MS/MS spectrum of the Su(H) phosphopeptide LRpSQTVSTR (precursor ion m/z = 564.2816, z = 2). Identity and sequence of the peptide as well as the phosphorylation site at S269 were confirmed by b- and y-ion series as indicated in blue and red, respectively. Neutral loss reactions of H_2_O and H_3_PO_4_ from the precursor ion are indicated in green. (**b**) Scheme of the wild type Su(H) protein [Su(H)^wt^], consisting of three domains: NTD (N-terminal domain; AS 116–252, light blue), BTD (beta-trefoil domain; AS 253–400, purple) and CTD (C-terminal domain; AS 424–516, dark blue). Below, the sequence of a BTD segment overlapping the phospho-peptide isolated by mass spectra is shown [underlined, bold in *D*. *melanogaster* Su(H)]; it is completely conserved in *H*. *sapiens* CBF-1, *M*. *musculus* RBPJ and *C*. *elegans* Lag1 proteins. Yellow triangles mark amino acids that contact the DNA backbone, light yellow triangles indicate base contacts via hydrogen bonds^22,28^. Ser269 is highlighted in red. The phospho-deficient [Su(H)^S269A^], the phospho-mimetic [Su(H)^S269D^] and the DNA-binding deficient [Su(H)^R266H^] mutant isoforms are indicated. Su(H)^R266H^ corresponds to the naturally occurring *Su(H)* ^*S5*^ mutant^24^. (**c**) Structure of Su(H) bound to DNA [PDB ID: 5E24]. Enlargement of the BTD-DNA contact is shown; Ser269 and Arg266 are highlighted. Figure was assembled using PyMOL, licensed to RAK.

The previous finding of a MAPK-site in the CTD of Su(H)^19^ sparked our interest to search for further phosphorylation sites in Su(H) in order to identify additional cross-talk between Notch and as of yet unknown signalling pathways. To this end, we took a mass spectrometry approach and isolated Su(H) protein from *Drosophila* Schneider S2 cells. Myc-tagged Su(H) protein and activated Ras^V12^ were ectopically induced in S2 cell culture followed by immunoprecipitation of Su(H) protein with anti-Myc antibodies. The upper of two Su(H) protein bands was excised and in-gel digested with trypsin (Fig. 1a). Resultant peptides were analyzed by nano-LC-ESI-MS/MS with a verified sequence coverage of 38% of the Su(H) protein. A singly phosphorylated peptide (LRpSQTVSTR) corresponding to amino-acids 267–275 of Su(H) was identified by MS/MS analysis (Fig. 1a). The fragmentation spectrum of the phosphopeptide showed a good sequence coverage by y- and b-ions, enabling an unambiguous localization of the phospho-site to Serine 269 (Fig. 1a).

The position of this newly identified phosphorylation site resides within the beta-trefoil domain (BTD) of Su(H), which is known to interact with the RAM domain of the intracellular domain of Notch (NICD) and to mediate contact with DNA (Fig. 1b,c)^20–22^. Therefore, phosphorylation at this site might influence the high-affinity interaction with NICD, interfering with activator complex formation. Similarly, Nemo-like kinase (NLK) inhibits ternary complex formation by phosphorylation of NICD^23^. Alternatively, phosphorylation might generally affect the binding of Su(H) to target sites on the DNA. Ser269, as well as the sequence surrounding it, is absolutely conserved between *Drosophila*, *C*. *elegans* and mammals, including humans, and lies within a region known to make both specific and nonspecific contacts with the DNA (Fig. 1b,c)^20^. To address the potential biological significance of Ser269 phosphorylation we generated two mutants by site directed mutagenesis: a phospho-deficient Su(H) isoform by replacing Ser269 with alanine (S269A), and a phospho-mimetic Su(H) isoform by replacing Ser269 with aspartic acid (S269D) (Fig. 1b). In addition, we replaced Arg266 by Histidine (R266H), thereby re-creating a DNA binding-deficient Su(H) protein, also known as Su(H)^S5^ mutant^24^ (Fig. 1b). The mutant constructs were shuttled into respective vectors to allow for subsequent analyses *in vitro* and *in vivo*.

### The phospho-mimetic Su(H)^S269D^ isoform shows reduced activity in *Drosophila* cells

**Figure 2:**
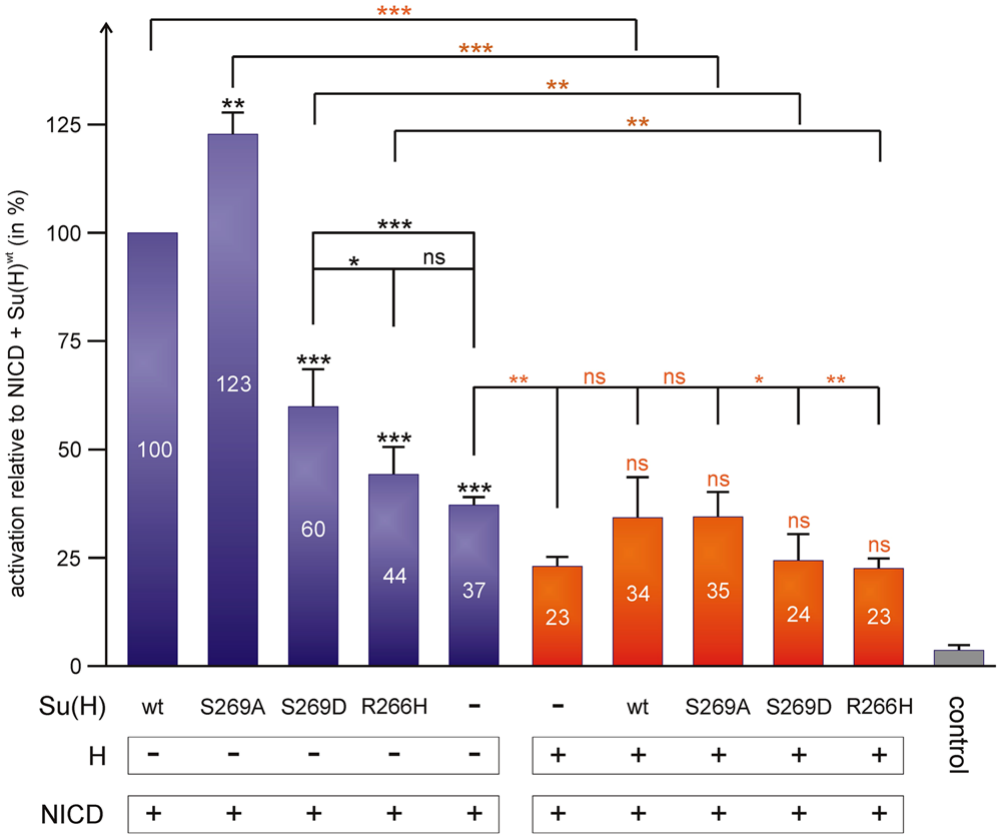
Notch signalling readout is changed in S2 cell culture assays. S2 cells were co-transfected with the NRE luciferase reporter construct and with NICD to induce expression plus the different Su(H) constructs as indicated. Reporter activity in cells co-transfected with Su(H)^wt^ and NICD was taken as 100%. Co-transfection with Su(H)^S269A^ significantly increases reporter activity (about 120%), whereas co-transfection with either Su(H)^S269D^ or Su(H)^R266H^ had a much lower effect on NICD activity. Repressor complexes were tested with a co-transfection of NICD with H and respective Su(H) variants (orange bars). H inhibits ICN-mediated reporter-gene activation down to about 60%. This inhibition is alleviated by the combined co-transfection of NICD and H with either Su(H)^wt^ or Su(H)^S269A^: apparently the two behave similarly in this context. Co-transfection of NICD and H with either Su(H)^S269D^ or Su(H)^R266H^ show the same low reporter gene activation as NICD and H alone: both seem incapable of interfering with H-mediated repression. Three independent assays each were performed, measurements were in duplicate. Error bars denote standard deviation; ***p < 0.001 highly significant; **p < 0.01 very significant; *p < 0.05 significant; p > 0.05 not significant (ns). Symbol on top of the bars represent comparison to control, to either NICD + Su(H) (blue columns) or NICD + H (orange columns), respectively. Other comparisons are as indicated by horizontal lines.

First, we addressed the transcriptional activity of the phospho-mutant Su(H) isoforms in *Drosophila* Schneider S2 cells by transient transfection with respective inducible constructs. As a read out, luciferase activity was measured from a Notch response element reporter construct (NRE-luc)^25^. NRE-reporter gene activity is induced by transfection of NICD, which can be further raised about threefold by co-transfection with wild type Su(H)^wt^ (Fig. 2)^12,25,26^. Compared to the level of Su(H)^wt^, the phospho-deficient Su(H)^S269A^ mutant construct resulted in a slightly enhanced activation, whereas Su(H)^S269D^ resulted in a considerably lower reporter activation. As expected, the Su(H)^R266H^ mutant had no impact on the NICD-mediated activation of the NRE-luc reporter (Fig. 2, compare lanes 4 and 5). We conclude that phosphorylation of Su(H) at Ser269 impedes Notch activity, which is not a consequence of a mutation of Ser269 per se, since Su(H)^S269A^ shows an even stronger activity than wild type. Moreover, as our mass spec data shows that Su(H) is phosphorylated in S2 cells, the increased activity of Su(H)^S269A^ suggests that repression of Notch signalling activity by phosphorylation is in fact taking place in these cells even in the absence of activated Ras^V12^.

The Su(H) transcriptional regulator not only activates, but also represses Notch target genes, depending on its bound co-factors. We therefore wanted to determine whether repression activity was likewise affected in the Su(H)^S269D^ mutant. To this end, a co-transfection of the respective constructs with NICD and Hairless (H) was performed. Transfection of H results in a repression of NICD-mediated reporter gene activity by about two-fold^26^. In combination with either wild type Su(H)^wt^ or Su(H)^S269A^ values are similar to NICD overexpression alone. In contrast neither Su(H)^S269D^ nor Su(H)^R266H^ had a significant effect on H-mediated repression (Fig. 2). Although compensation of H overexpression by Su(H)^wt^ and Su(H)^S269A^ was not significant in this assay, the tendency of differential activity is apparent. Hence, we propose that phosphorylation of Su(H) at Ser269 may mitigate transcriptional activity, affecting both activation and repression of Notch target genes alike.

### The phospho-mutant Su(H) isoforms are not impaired in protein complex formation

**Figure 3:**
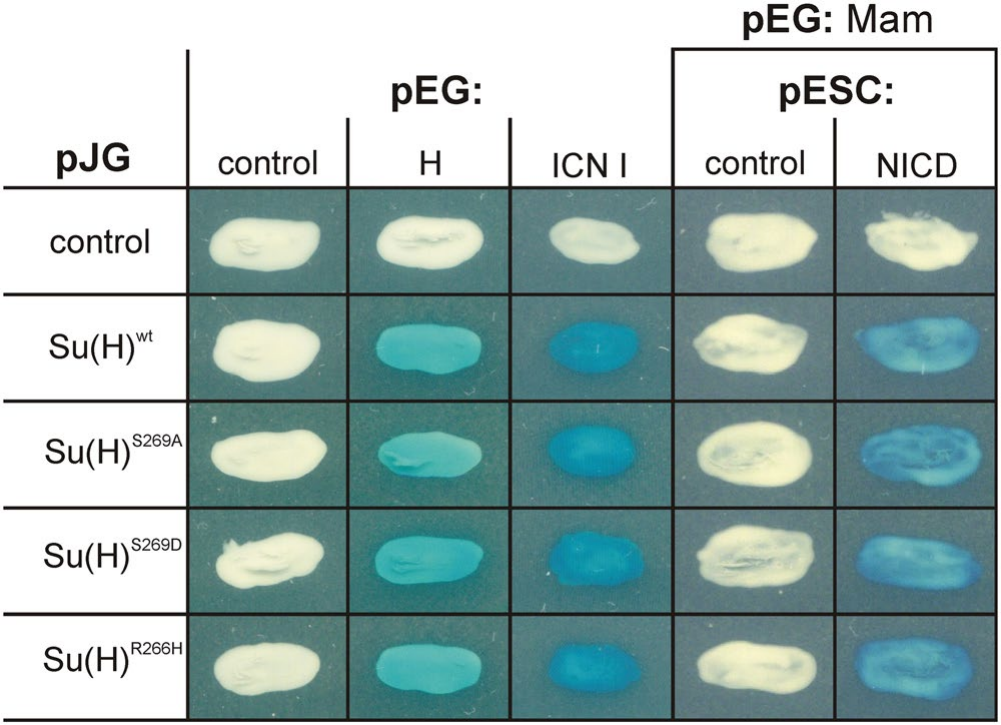
Yeast two- and three hybrid interaction assays. Direct protein-protein interaction between the Su(H) variants (in pJG) and either full length H or the intracellular part of the N receptor (ICN I, in pEG), were tested in a yeast two-hybrid assay. Interaction is seen by the blue colour resulting from the activation of the reporter gene lacZ, whereas white yeast colonies indicate no interaction. Yeast three-hybrid tests^12,13^ were done with pEG-Mam and pESC-NICD transformed with the different Su(H) variants in pJG vector, respectively. Empty vectors served as mock control.

The reduced activation and repression activity of Su(H)^S269D^ in the above luciferase assay could result from a failure to assemble activator complexes together with NICD and Mam, or repressor complexes together with H. To assay protein complex formation of the Su(H) mutant isoforms, yeast two- and three-hybrid assays were performed, addressing the direct interaction of Su(H) with either H or NICD, as well as the assembly of the Notch activator complex, which includes Mam. As shown in Fig. 3, all mutant isoforms, the phospho-specific Su(H)^S269A^ and Su(H)^S269D^ mutants as well as the DNA binding-deficient Su(H)^R266H^ mutant proteins, performed very similar to wild type Su(H) in the binding of either H or NICD, or in the three-hybrid assay together with NICD and Mam. These results imply that phosphorylation of Su(H) at Ser269 does not interfere with the assembly of protein complexes. We next addressed the possibility that the mutations might affect protein expression, stability or nuclear localization of Su(H) protein. To this end, expression of the Su(H) isoforms was induced by a heat pulse and detected 6 hours later on Western blots. We observed little variance in the levels of the induced proteins (Supplementary Fig. S1). Hence the observed differences in Su(H) activity are unlikely to result from differences in expression or stability. Moreover, nuclear localization was observed for the phospho-mutant Su(H) variants when induced in S2 cells (Supplementary Fig. S1). In sum it appears more likely that the DNA-binding activity of Su(H) is altered by the phosphorylation, presumably by adding a negative charge in the vicinity of the residues that are directly contacting DNA (Fig. 1c).

### Altered DNA binding of phospho-mutant Su(H) proteins

**Figure 4:**
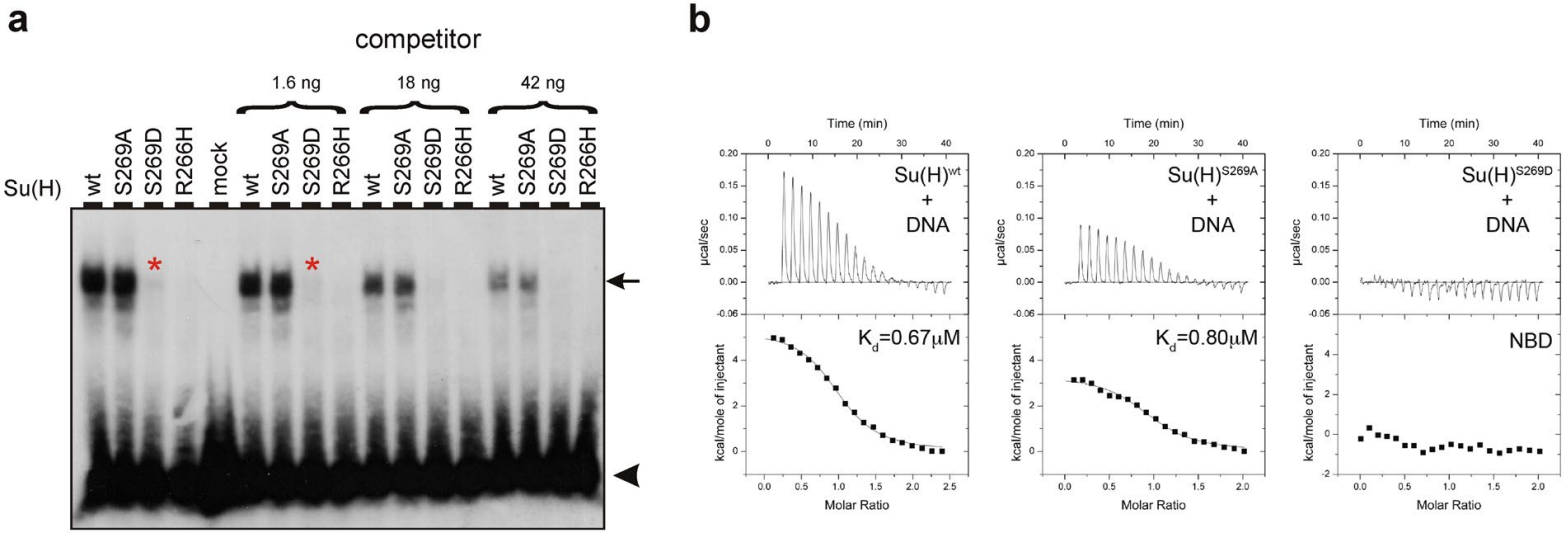
DNA binding is affected in the phospho-mimetic Su(H)^S269D^ isoform. (**a**) Electro-mobility shift assay for the binding of the indicated Su(H) isoforms to radiolabelled E(spl)m8-S1 oligo^27^. In the mock control, no protein was added to the reaction. Su(H)^wt^ and likewise Su(H)^S269A^ bind well, whereas only a very weak binding of Su(H)^S269D^ (red asterisks) and no binding of Su(H)^R266H^ to the oligo was detected. Specificity was tested by adding increasing amounts (1.6 ng, 18 ng, 42 ng) of unlabelled competitor oligo. The arrow points to the shifted complexes; the arrowhead demarks the radiolabelled unbound oligos. (**b**) Isothermal titration calometry assay of Su(H)-DNA binding. Representative thermograms for Su(H) binding to DNA comprising the consensus HES-1 site are shown (raw heat signal and nonlinear squares fit to the integrated data). Twenty titrations were performed per experiment, consisting of 14 μl injections that were spaced 120 s apart.

To directly address the DNA binding properties of the Su(H) protein isoforms, wild type as well as the two phospho-specific mutants were *in vitro* transcribed and translated, and subjected to an electro-mobility shift assay (EMSA) using a radiolabelled E(spl)m8-S1 oligo as target DNA^27^ (Fig. 4a, Supplementary Fig. S2). For comparison, the defined DNA binding deficient Su(H) mutant isoform Su(H)^R266H^ was also used^24^. Whereas no obvious difference in DNA binding was observed between wild type and the phospho-deficient isoform Su(H)^S269A^, the phospho-mimetic Su(H)^S269D^ isoform was strongly impaired in DNA binding. As expected, DNA-binding was completely abrogated in the Su(H)^R266H^ mutant (Fig. 4a). However, in contrast to Su(H)^R266H^, we were able to observe very weak DNA binding by Su(H)^S269D^ (Fig. 4a, red asterisk). In order to quantify the differences we performed isothermal titration calorimetry (ITC) with recombinantly purified Su(H) protein and oligomeric DNA duplexes that contain the consensus binding site of the HES-1 gene^28^. The ITC experiments revealed little differences between the energetics of either Su(H)^wt^ or Su(H)^S269A^ – DNA binding (Fig. 4b, Supplementary Fig. S2). In contrast to our EMSA results, we could not detect any DNA binding of Su(H)^S269D^ by ITC (Fig. 4b, Supplementary Fig. S2). These differences are likely a result of the differences in detection limits between the two methods. From these results we conclude that phosphorylation at Ser269 interferes with the DNA binding capacity of the transcription factor Su(H). This could mean that, depending on the context of such a phosphorylation, either the activation of Notch target genes or their repression is downregulated by an unknown kinase. For example, if this kinase is activated concurrent with the Notch receptor, Notch target gene activation would be inhibited. If the kinase, however, is active whilst Notch signalling is off, Notch target gene repression would be alleviated.

### ***In vivo*** studies of phospho-mutant Su(H) variants during wing imaginal development

**Figure 5:**
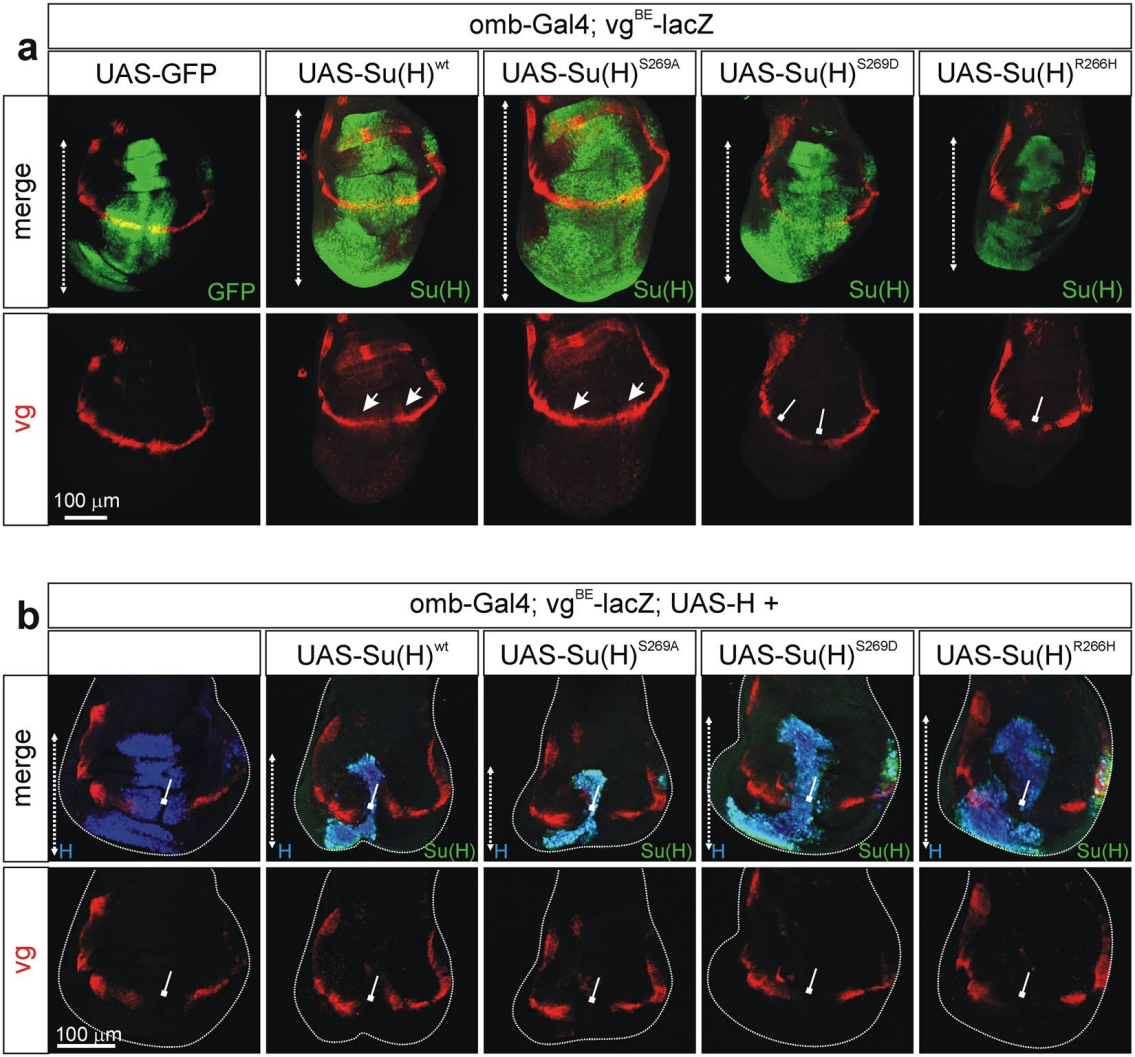
Effect of phospho-mutant Su(H) variants on Notch target gene *vestigial* expression. (**a**) *vg* ^*BE*^-*lacZ* reporter activity along the dorso-ventral boundary of the wing imaginal disc was detected with anti-beta-Galactosidase antibodies (red). Su(H) isoforms as indicated (shown in green) were induced in the central part of the wing disc anlagen with the *omb*-Gal4 driver. As control UAS-GFP (green) was overexpressed. Overexpression of UAS-Su(H)^wt^ or UAS-Su(H)^S269A^ caused overproliferation in the affected tissue (double headed arrow) and enforced *vg* ^*BE*^ *-lacZ* activity at the dorso-ventral boundary (arrows). In contrast, overexpression of UAS-Su(H)^S269D^ reduced *vg* ^*BE*^ *-lacZ* expression (repressive beam) and had little effect on tissue size. A similar phenotype was seen after overexpression of UAS-Su(H)^R266H^. (**b**) Overexpression of UAS-H (shown in blue) using *omb*-Gal4 represses *vg* ^*BE*^ *-lacZ* reporter activity (red) at the dorso-ventral boundary (repressive bar). Co-overexpression with the indicated UAS-Su(H) constructs (shown in green; appears turquoise in the combination with UAS-H): wild type and phospho-deficient Su(H) enhances H-mediated repression, leading to a strong reduction of vg expression (repressive bars) and a further decrease in size of affected tissue. The phospho-mimetic and the DNA-binding deficient Su(H) isoforms had little effect on H-overexpression phenotypes. This is especially apparent when comparing tissue size (double headed arrow). Size bar represents 100 μm in all panels.

With the goal to examine the *in vivo* consequences of Ser269 phosphorylation, the Su(H) mutant constructs (Fig. 1b) were cloned under UAS-control, enabling us to induce their expression in a tissue specific manner in transgenic flies. Position effects were avoided by using the PhiC31 integrase system that allows insertion of each mutant construct at the identical chromosomal position^29^. Ectopic expression of the different Su(H) mutant variants and the wild type form was induced with *omb*-Gal4 in the central wing anlagen. Notch *in vivo* activity was monitored with the *vg* ^*BE*^ *-lacZ* reporter line, which is induced in a stripe along the dorso-ventral boundary of the presumptive wing blade (Fig. 5a)^30^. In addition, the expression of Cut protein served as readout of Notch activation along the dorso-ventral boundary^31^ (Supplementary Fig. S3). In this context, overexpression of wild type Su(H) causes a slight expansion of *vg* ^*BE*^ *-lacZ* or of Cut expression^12,13,26^, which was likewise observed upon overexpression of the phospho-deficient Su(H)^S269A^ isoform (Fig. 5a; Supplementary Fig. S3). In accordance with a gain of Notch activity^32,33^, the wing discs were overgrown, again with little variance between Su(H)^wt^ and Su(H)^S269A^ (Fig. 5a; Supplementary Fig. S3). In contrast, overexpression of Su(H)^S269D^ caused a patchy expression of *vg* ^*BE*^ *-lacZ* and a strong repression of Cut, but had little effect on the size of the wing disc (Fig. 5a, Supplementary Fig. S3). Apparently, Su(H)^S269D^ was not only impaired in its ability to activate Notch mediated reporter expression, but instead caused its repression. The latter might be a consequence of Su(H)^S269D^-NICD protein binding and simultaneous failure of DNA binding, i.e. dysfunctional activator complexes are formed that cannot localize to Notch target genes. Hence, overexpression of Su(H)^S269D^ acts in a dominant negative fashion on activated NICD molecules. Accordingly, overexpression of the Su(H)^R266H^ variant – which completely fails to bind to DNA *in vitro* – showed an even stronger repression of *vg* ^*BE*^ *-lacZ* or Cut, and also no increase in disc size (Fig. 5a, Supplementary Fig. S3).

Due to its role as a molecular switch, Su(H) is required for both Notch target gene activation and repression. With the goal to analyse the ability of the different phospho-specific Su(H) variants during repression, co-expression experiments with H were performed. Overexpression of H interferes with many Notch-dependent processes during larval development, for example the formation of the peripheral nervous system like mechano-sensory organs or during wing development (for review^14^). As H directly competes with Notch for interacting with Su(H) protein, gain of H equalizes loss of Notch activity. Accordingly, overexpression of H results in repression of Notch target gene expression, which can be visualized with either *vg* ^*BE*^ *-lacZ* or Cut, and is accompanied by a reduction of tissue size^32^ (Fig. 5b; Supplementary Fig. S4)^26^. A combined overexpression of H and Su(H) results in an extreme inhibition of Notch activity as a result of excessive repressor complex formation^12,13,34^. As a consequence many of the affected cells are lost presumably by apoptosis^35–37^, and only a small remainder of the expression domain is observed accompanied by a total repression of Notch target gene expression (Fig. 5b; Supplementary Fig. S4). A very similar result was seen when H is overexpressed together with Su(H)^S269A^, indicating that a mutation of Ser^269^ per se does not interfere with Su(H) activity. A considerably weaker phenotype was seen upon combined overexpression of H with Su(H)^S269D^, and even more so with Su(H)^R266H^ (Fig. 5b; Supplementary Fig. S4). These results show that repressor complex assembly itself is not affected, but rather these complexes are less active, which can be attributed to the impairment of DNA binding. Dominant negative effects are not apparent in the combined overexpression, as one might have expected a size increase of tissue relative the H overexpression alone (Supplemental Fig. S4). This might be explained by the fact, the Su(H) mutant proteins interfere with activated Notch at the same time, contributing to a loss of Notch activity in addition to impeding H. Overall, these *in vivo* data support the results from our cell culture studies, indicating that phosphorylation of Ser^269^ is equivalent to a deficit of Su(H) activity as a consequence of lowered DNA binding.

### ***In vivo*** studies of phospho-mutant Su(H) variants in the adult fly

**Figure 6:**
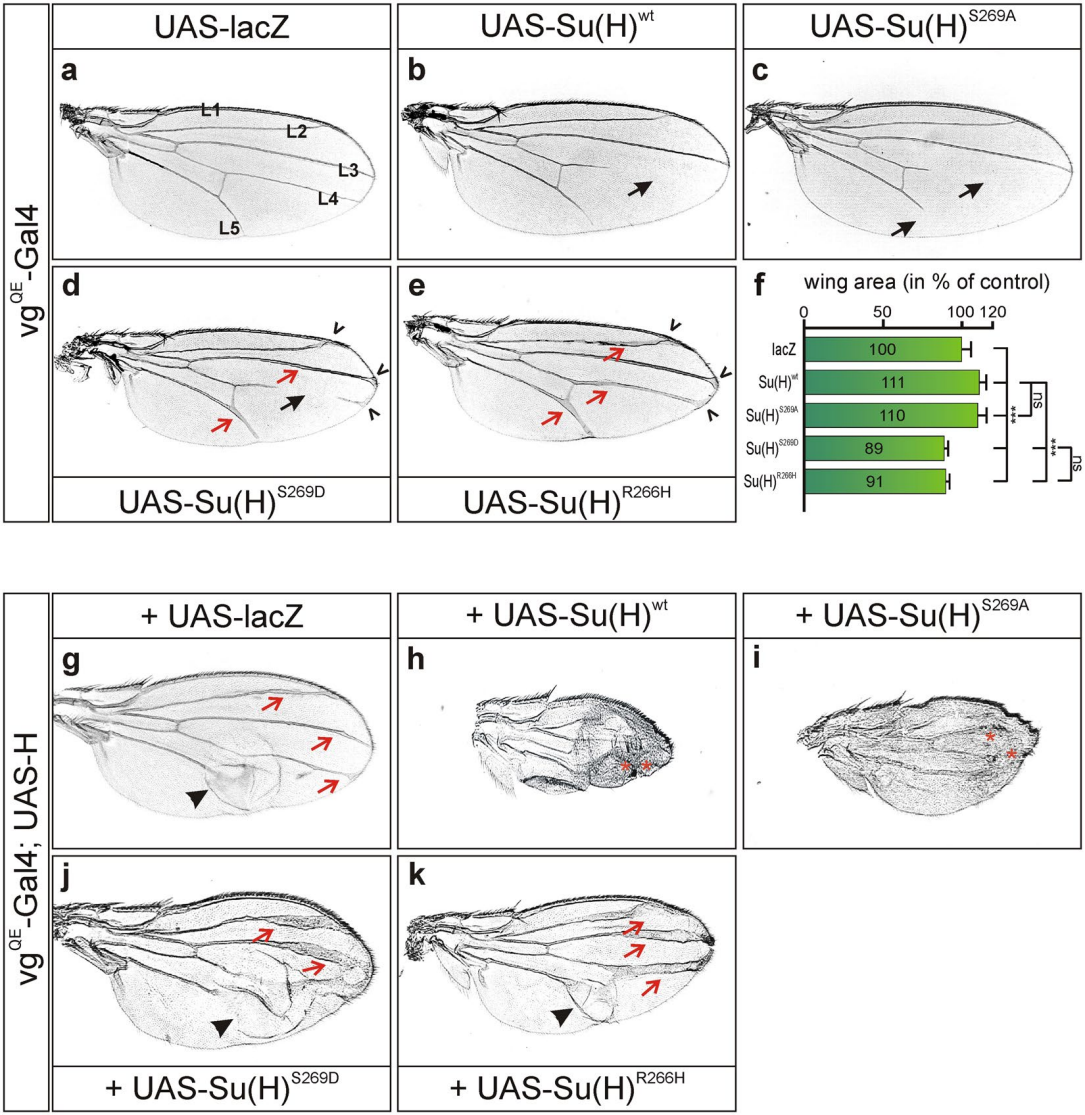
Consequences of ectopic expression of phospho-mutant Su(H) variants on adult wings. Overexpression of the indicated UAS-constructs was induced in the distal wing anlagen with the *vg* ^*QE*^-Gal4 driver; (**a**–**f**) shows Su(H) variants alone and (**g**–**k**) in combination with full length H. Representative wings from female flies are shown. (**a**) UAS-lacZ was used as control. Longitudinal veins L1-L5 are indicated. (**b**) Overexpression of *Su(H)* ^*wt*^ and (**c**) Su(H)^S269A^ causes gaps in the longitudinal veins L4 and L5 (black arrows). In contrast, overexpression of (**d**) *Su(H)* ^*S269D*^ or (**e**) *Su(H)* ^*R266H*^ results in thickened veins (red arrows) and deltas at the wing margin (open arrowheads) indicative of a reduced Notch signalling activity. In some cases also gaps can be detected within the same wing (arrow; example is shown in (**d**). (**f**) Measurements of the area from 8 female wings overexpressing the given UAS-Su(H) construct. *Su(H)* ^*wt*^ and *Su(H)* ^*S269A*^ overexpression increased wing size, whereas that of *Su(H)* ^*S269D*^ or *Su(H)* ^*R266H*^ reduced it significantly relative to control. Error bars represent standard deviation (n = 8); ***p < 0.001 highly significant; p > 0.5 not significant (ns). **(g)** Wings derived from a combination of UAS-H plus UAS-lacZ served for comparison with UAS-H plus any of the different UAS-Su(H) variants, to account for the titration effect of Gal4. Crosses were kept at 18 °C to circumvent early lethality. Compared to the control UAS-lacZ (**a**), UAS-H (**g**) causes thickened veins (red arrows) and blistered wings (arrowhead) when induced with *vg* ^*QE*^-Gal4. This effect is strongly enhanced by a concomitant overexpression of H with either (**h**) *Su(H)* ^*wt*^ or (**i**) *Su(H)* ^*S269A*^ resulting in tiny wings where large parts are completely transformed into vein material, recognized by more densely spaced cells and ectopic bristles (red asterisks). This enhanced phenotype was not observed when H was co-overexpressed with either (**j**) *Su(H)* ^*S269D*^ or (**k**) *Su(H)* ^*R266H*^. Yet, distal veins were conspicuously thicker (red arrows) in these combinations [compare (**j**) and (**k**) with (**g**)].

Next we wanted to visualize the consequences of an overexpression of the phospho-mutant Su(H) variants on fly development. This is hampered by the fact that a manipulation of Notch signalling activity during larval development most often results in larval or pupal lethality, limiting the processes and tissues to be analysed. Lateral inhibition is one of the best-studied processes mediated by the Notch pathway, a process in which a few cells are selected from a group of equipotent cells to assume the primary cell fate, whereas the others are forced into a secondary fate (for review^5–7^). For example, during wing vein development Notch signalling is required to restrict vein competence, thereby limiting the width of the vein to its normal size^38^. We used this process to address the activity of the various Su(H) isoforms. Using *vg* ^*QE*^-Gal4, the respective UAS-constructs were overexpressed exclusively within the presumptive wing blade, sparing the morphogenetic antero-posterior and dorso-ventral boundaries^19,30^. Both, Su(H)^wt^ and Su(H)^S269A^ overexpression caused a loss of vein material, in agreement with an enhancement of vein fate restriction due to increased Notch activity. Notably, the distal parts of the longitudinal L4 and L5 veins were affected (Fig. 6a–c), a phenotype indistinguishable from a heterozygous *H* null mutant fly, which also has an increase in Notch activity^39,40^. Notch gain of function phenotypes resulting from Su(H) overexpression can be largely explained by a Su(H)-H titration effect. In fact, overexpression of just the Su(H) CTD, which is capable of binding to H but is itself inactive in Notch signal transduction, is sufficient to induce Notch gain of function phenotypes^41^. One might have expected a similar increase in Notch activity when overexpressing Su(H)^S269D^ or Su(H)^R266H^ since both are able to titrate H. This was not observed. In contrast, overexpression of Su(H)^S269D^ and Su(H)^R266H^ caused predominantly thickened veins and deltas at the margin (Fig. 6d,e), a typical consequence of a Notch loss of function, resulting from a failure to restrict vein width^38^. As both two Su(H) isoforms are hampered in DNA-binding, any activator complex formed together with NICD will be non-functional, i.e. both isoforms also titrate active Notch, providing an explanation for the observed phenotypes. In addition to thickened veins, overexpression of Su(H)^S269D^ also caused distinct vein gaps in the longitudinal vein 4 (Fig. 6d), indicative of a gain of Notch signalling activity. This mixed phenotype, i.e. increased vein thickness and vein loss, may be explained by the observation that Su(H)^S269D^ has somewhat residual DNA binding capacity, allowing little signal transduction and hence, some of the expected gain of Notch phenotypes. In addition to vein thickening, we also noted a conspicuous change in the wing size: an enlargement when overexpressing either Su(H)^wt^ or Su(H)^S269A^, and a decrease when inducing Su(H)^S269D^ or Su(H)^R266H^, reflecting primarily gain of Notch activity in the former two and loss in the latter (Fig. 6f).

To follow the effects of the phospho-mutant Su(H) isoforms exclusively on the repression of Notch signalling activity, we simultaneously overexpressed H within the wing anlagen. As expected, sole overexpression of H with *vg* ^*QE*^ caused a broadening of the longitudinal veins (Fig. 6g), a phenotype which was strongly enhanced when Su(H) or Su(H)^S269A^ were induced at the same time: the wings were extremely small and much of the tissue was directed to vein fate (Fig. 6h,i). In contrast, the wings obtained after co-overexpression of H with either Su(H)^S269D^ or Su(H)^R266H^ resembled those resulting from the sole H overexpression with regard to size and residual intervein material, although the distal portions of the longitudinal veins were clearly thicker (compare Fig. 6j,k with g).

## Discussion

In this study we identified phosphorylation of Su(H) at Serine 269 by nano-LC-ESI-MS/MS and provide evidence for the function of this modification *in vivo*. A phospho-mimetic mutation of this site [Su(H)^S269D^] results in strongly reduced DNA binding, which is accompanied by a mitigation of Notch signalling readout in several different developmental contexts tested. As Su(H) acts as a switch in the Notch pathway, being the nuclear effector for both activator and repressor complexes, it follows that Su(H)^S269D^ could either affect the transcriptional activation or the repression of Notch target genes, depending on the cellular context. If Su(H) phosphorylation occurred simultaneously with Notch receptor activation, transcriptional activation would be lessened by the reduced DNA binding affinity of Su(H). At other times, however, Su(H) phosphorylation would result in a release of target gene inhibition, since binding of the Su(H)-H repressor complex would be mitigated. Consistent with this premise, our overexpression studies demonstrate that both, transcriptional activation and repression are affected *in vivo*. Bearing the limitations of overexpression studies in mind, the respective contribution of Ser 269 phosphorylation during normal development and signalling situations, however, remains to be determined.

Structure analyses revealed that CSL transcription factors recognize their cognate DNA binding site [C/tGTGGGAA] through extensive sequence-specific and non-specific DNA contacts mediated by the BTD and NTD^20^. The NTD makes major-groove sequence specific contacts to the second half of the binding site (5′-GGGA-3′) in a manner similar to other Rel-DNA complexes. The BTD also makes specific DNA contacts via a beta-hairpin loop that inserts into the minor-groove sequence, thereby selecting for the 5′ pyrimidine followed by a G (5′-C/t G)^20^. CSL-DNA crystal structures have been determined from human, mouse, *C*. *elegans* and *Drosophila* proteins with DNA corresponding to the consensus HES-1 binding site, and remarkably, the structures are very similar despite the huge evolutionary distance of the studied species (for review^11^). Interestingly, the beta-hairpin loop of the BTD involved in DNA contacts appears to be flexible as it can adopt alternate conformations^28,42^: in the preferred conformation the backbone carbonyl of Ser269 and the side chain of Gln270 make sequence specific contacts [numbering is from *Drosophila* Su(H)]. In alternative conformations, however, Ser269 can move into the place of Gln270 to make side chain-hydrogen bonds^28^. Alanine replacement of Ser269 should likewise allow for the backbone carbonyl contacts but not for side chain-hydrogen bonds, which would likely have little impact on Su(H)-DNA complex stability, but may affect specificity. Whereas aspartate at this position is expected to interfere with DNA binding altogether due to its negative charge. Perhaps the flexibility of the beta hairpin-loop is an important aspect of phospho-Ser269 regulation, since it is conceivable that its conformation influences the accessibility of Ser269 by protein kinases. This flexibility may also underlie the molecular basis for why Su(H)^S269D^ retains some DNA binding in our EMSA data, whereas Su(H)^R266H^ does not. Moreover, there are indications that the conformation of the BTD beta-hairpin loop may correlate with target sequences bound by CSL^28^. In this case, Ser269 phosphorylation of Su(H) might be target gene specific and would result in a specific rather than a general downregulation of Notch signalling activity within a cell.

Our new results uncover extensive crosstalk between the Notch signalling pathway and other signalling cascades by means of phosphorylation. They reveal a second example of CSL phosphorylation. Earlier, we have shown that Su(H) is targeted within its C-terminus at Thr426 by MAPK, resulting in an attenuation of Notch signalling readout^19^. Also the MAPK-target site is absolutely conserved between fly and vertebrates. In mouse, phosphorylation of RBPJ at the corresponding Thr339 by the stress kinase p38 MAPK results in a destabilization of RBPJ^43^. In *Drosophila* we have, however, no indications to date that phosphorylation regulates Su(H) protein stability, despite the fact that Su(H) protein stability is an important means of regulating its availability^44^. The relative protein levels of either Su(H)^wt^, Su(H)^S269D^ or Su(H)^S269A^ were very similar after overexpression. If phosphorylation at this site resulted in a destabilisation or even degradation of the protein, we might have expected to see differences in stability. There is ample evidence for the influence of phosphorylation on the stability and the half-life of a protein. A prime example is the Notch receptor itself, as NICD is phosphorylated and degraded upon signalling^45^.

According to computational predictions based on the phosphorylation site database algorithm (PHOSIDA) several kinases may be considered to recognize Ser269 as target site^46^. Although we have identified the phosphorylation of Ser269 in cultured S2 cells in the presence of activated Ras^V12^, the sequence surrounding Ser269 does not have a typical MAPK signature. Perhaps phosphorylation of Ser269 is mediated by a kinase, which itself is activated upon high levels of Ras signalling. As active Ras controls key aspects of growth, cell death, metabolism and mediates inter-cellular stress responses^47^ it might also pilot Notch signalling activity via Su(H) phosphorylation by an as yet unknown kinase. Alternatively, Ser269 phosphorylation may be independent of Ras activity. To our knowledge, no murine or human homologue phosphorylated at the corresponding Ser has been identified in high throughput screens (http://www.phosphosite.org/). It is conceivable that Ser269 in Su(H) is phosphorylated constitutively, and dephosphorylated specifically by an unknown phosphatase in a context specific manner. As this phosphorylation target site is absolutely conserved in all mammalian CSL orthologues it is tempting to speculate that – as in *Drosophila* - this site is used for crosstalk in mammals as well, opening the possibility for a new strategy to achieve tissue homeostasis and to consequently coordinate and react on cellular stress signals.

## Methods

### Plasmid construction, generation of transgenic flies and fly work

Su(H) cDNA in Bluescript vector was used for QuickChange site-directed mutagenesis (Agilent, La Jolla CA, USA) to generate Su(H)^S269A^, Su(H)^S269D^ and Su(H)^R266H^. Sequence verified constructs were cloned as *Acc*65I/*Xba*I fragments into the pUAST-attB vector. Transgenic flies were established with the PhiC31 integrase-based system^29^ using the ΦX-58A or ΦX-96E strain harbouring the landing site at 58A and 96E, respectively. Correct integration was tested by PCR. Other fly stocks used: *omb* ^*md65*^-Gal4/FM7^48^, *vg* ^*QE*^-Gal4^19^, *vg* ^*BE*^-lacZ^30^, Sco/CyO hs-Gal4 UAS-GFP (BL5702), UAS-*GFP* (BL4776), UAS-*lacZ* (BL8529), UAS-*H* at 68E^12^. For co-overexpression experiments UAS-*Su(H)* wild type^12^ or mutant constructs at 96E were recombined with UAS-*H* at 68E. Flies were raised on standard cornmeal at 18 °C and crosses kept at 18 °C or 25 °C as indicated.

### Mass spectrometry

Myc-tagged Su(H) protein was derived from stably transfected Schneider S2 cells by immuno-precipitation as described before^19^. The coomassie stained Su(H) protein was in-gel-digested with trypsin and mass spectrometry analysis was performed on a Thermo LTQ-Orbitrap instrument (Thermo Fisher Scientific, Germany) equipped with a nanoelectrospray ion source as described before^49^. Survey spectra (m/z = 300–1800) were detected in the Orbitrap at a resolution of 60,000 at m/z = 400. Database searches were performed with MASCOT search algorithm to identify the Su(H)-phosphopeptide, verified by manual inspection of the MS/MS spectra^49^.

### Yeast two-hybrid constructs and assays

Yeast two- and three-hybrid experiments were performed as described before^12,13,35^. The following constructs were used: pEG-ICN I (codons 1827–2259)^50^, pEG-Mam (codons 118–194), pESC-NICD (codons 1762–2176)^13^, pEG-H (full length Hairless, corresponding to pEG-HFL)^51^. The Su(H)^S269A^, Su(H)^S269D^ and Su(H)^R266H^ mutant variants were cloned as full length constructs into pJG vector^52^ and were sequence verified.

### S2 cell culture and transfection assays

Schneider S2 cell culture reporter assays were performed at least in triplicate according to ref.^12^. Cells were transiently transfected with 1 μg NRE-luciferase reporter^25^ and 0.2 μg of control Renilla plasmid for normalization. cDNA of Su(H)^wt^, Su(H)^R266H^, Su(H)^S269A^ and Su(H)^S269D^ was cloned as 1.8 kb *Eco* RI/*Kpn* I fragment into pRmHa3 (pMT) vector^53^. In addition, the constructs were tagged C-terminally with myc. Protein expression was induced with CuSO_4_ 6 h after transfection and luciferase activity was measured 18 h later using dual-luciferase reporter assay (Promega, Heidelberg, Germany) with Lumat LB 9507 (EG and Salem, MA, USA). Su(H) myc-tagged protein was precipitated from about 10^8^ Schneider S2 cells stably transfected with pMT-ras^V12^, pMT-Gal4 and UAS-Su(H)-myc^19^. The expression was induced with 0.7 mM CuSO_4_ and extracts were prepared 18-22 hours after induction.

### DNA binding studies

Electro mobility shift assays were performed as described before^19^ with a double stranded DNA-oligomer harbouring the E(spl)m8-S1 Su(H) binding site [5′-TTGGGTGGCTCGTGGCGTGGGAACCGAGCTGAAAG-3′, 5′-GGTTCTTTCAGCTCGGTTCCCACGCCACGAGCCAC-3′]^27^. Su(H) protein isoforms were produced by coupled *in vitro* transcription / translation with TNT-Coupled Reticulocyte Lysate System (Promega, Mannheim, Germany) and 5 µl used for the reaction. The binding buffer contained 10 mM Tris-HCl pH 7.5, 50 mM NaCl, 1 mM EDTA, 0.0275 µg/µl salmon sperm DNA.

For isothermal titration calometry (ITC), recombinant Su(H) (98–523) was overexpressed and purified from bacteria, as previously described^13^. ITC experiments were carried out using a MicroCal VP-ITC microcalorimeter exactly as outlined earlier using double stranded DNA with the Hes-1 consensus [5′-GTTACTGTGGGAAAGAAAG-3′, 5′-GGAAGTTTCCCACAGGCCG-3′]^28^. All Su(H)-DNA experiments were performed at 10 °C in 50 mM sodium phosphate pH 6.5 and 150 mM NaCl. Su(H) was degassed and buffer-matched using dialysis and size exclusion chromatography. A typical Su(H)-DNA binding experiment contained 10 μM Su(H) in the cell and 100 μM DNA in the syringe. The data were analyzed using ORIGIN software and fit to a one-site binding model.

### Immunochemistry and confocal imaging

Overexpression in wing anlagen was induced with *omb* ^*md65*^-Gal4^48^. Wing discs were isolated from late third instar larvae and stained as described before^54^. Expression of *vestigial* (*vg*) was determined using the *vg* ^*BE*^-*lacZ* reporter line^30^ and anti-β-galactosidase antibodies (DSHB, University of Iowa, Iowa City, IA, USA). For Western blots, hs-Gal4 was crossed to the respective UAS-Su(H) lines, and expression induced in embryos with one hour heat shock at 37 °C as described earlier^51^. Other antisera used were: anti-Cut (from DSHB, University of Iowa, Iowa City, IA, USA), anti-H-A^55^ or anti-Su(H) (Santa Cruz Biotechnology, Santa Cruz, USA). S2 cells transiently transfected with myc-tagged pMT-Su(H) constructs were stained as outlined before^56^ with anti-myc (Santa Cruz Biotechnology, Santa Cruz, USA), anti-Su(H)^39^ and anti-Pzg^57^ as nuclear marker. Secondary antibodies with minimal cross-reactivity coupled to DTAF, Cy3 and Cy5 generated in donkey were purchased from Jackson Immuno-Research Laboratories (Dianova, Hamburg, Germany). Discs were mounted in VectaShield (Vector Laboratories, California, USA) and documented with a Bio-Rad MRC1024 confocal system using LaserSharp 2000 imaging software coupled to a Zeiss Axiophot microscope (Carl Zeiss AG, Oberkochen, Germany). Pictures were assembled with Corel-PhotoPaint and CorelDRAW Version 9.0 software.

### Documentation of adult phenotypes

Adult wings were dehydrated in ethanol and mounted in Euparal (Roth, Karlsruhe, Germany), pictures of wings were taken with an ES120 camera (Optronics, Goleta, USA) using Pixera viewfinder software version 2.0. Wing size was measured with *ImageJ* programme.

### Statistical evaluation

Statistical significance of probes was evaluated by ANOVA using a two-tailed Tukey-Kramer approach for multiple comparisons (highly significant ***p < 0.001; very significant **p < 0.01; significant *p < 0.05; not significant (ns) p > 0.05).

### Data availability statement

All relevant data are within the manuscript and its supplementary information files.

## Electronic supplementary material

Supplementary Information
