# Supplementary material for: Phosphorylation of Suppressor of Hairless impedes its DNA-binding activity
Source: Sci Rep. 2017 Sep 19;7:11820. doi: 10.1038/s41598-017-11952-0 (PMC5605572; doi:10.1038/s41598-017-11952-0)
Supplement: Supplementary file 1 — Supplementary Information [file 41598_2017_11952_MOESM1_ESM.pdf]

Supplementary Information to

Phosphorylation of Suppressor of Hairless impedes its DNA-binding activity

Anja C. Nagel[1*], Jasmin S. Auer[1], Adriana Schulz[1], Jens Pfannstiel[2], Zhenyu Yuan[3], Courtney E. Collins[3], Rhett A. Kovall[3] and Anette Preiss[1]

[1] Institut für Genetik (240), University of Hohenheim, Garbenstr. 30, 70599 Stuttgart

[2] Core Facility Hohenheim, Mass Spectrometry Unit University of Hohenheim, 70599 Stuttgart

[3] Department of Molecular Genetics, Biochemistry and Microbiology, University of Cincinnati College of Medicine, Cincinnati, Ohio, United States of America.

* Corresponding author

e-mail: anja.nagel@uni-hohenheim.de

Supplementary Figure S1: Expression and nuclear localization of Su(H) variants

Supplementary Figure S2: Data supplementing Su(H)-DNA binding studies

Supplementary Figure S3: Effect of phospho-mutant Su(H) variants on Cut expression

Supplementary Figure S4: Consequences of combined overexpression of Hairless and phospho-mutant Su(H) variants on Cut expression

Supplementary Figure S1 Expression and nuclear localization of Su(H) variants

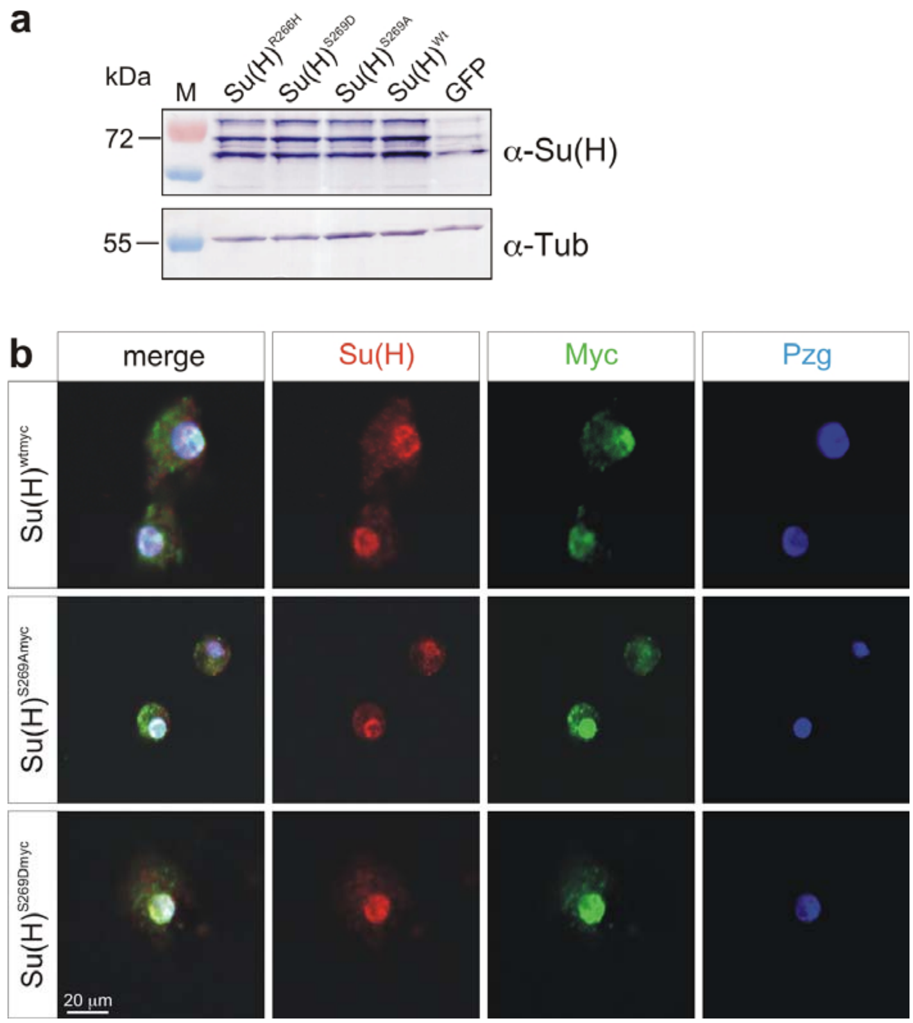

(**a**) Western blots of protein extracts derived from embryos that carried hs-Gal4 / +; UAS-Su(H)* /+ constructs (* indicating the given Su(H) variant); Su(H) protein expression was induced by a 1 hour heat pulse at 37°C; proteins were extracted 6 hours later. UAS-GFP served as control for endogenous Su(H) expression; beta-tubulin (Tub) as loading control. M, protein standard in kDa. Typically two Su(H) protein species are detected on Westernblots at around 70 kDa; the smallest one in this blot may result from degradation.

(**b**) S2 cells were transiently transfected with myc-tagged pMT-Su(H) constructs as indicated. Protein expression was monitored 12 hours later with antibodies against Su(H) (red) detecting also endogenous Su(H) protein, anti-myc (green) to detect the specific Su(H) variants, and Pzg (blue) as nuclear marker.

Supplementary Figure S2   Data supplementing Su(H)-DNA binding studies

a

competitor	0	1.6ng	18ng	42ng
Su(H)	100%	89.1%	67.4%	49.4%
Su(H)^{S269A}	100%	87.1%	64.9%	47.4%

b

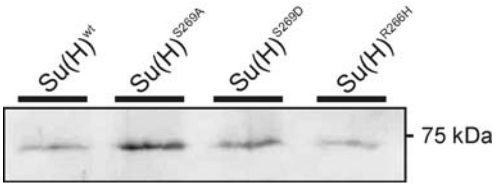

c

Su(H)	*K* (M^{-1})	K_d (μM)	*ΔG°* (kcal/mol)	*ΔH°* (kcal/mol)	*-TΔS°* (kcal/mol)
WT	1.49 ± 0.11 x 10^6	0.67 ± 0.05	-8.00 ± 0.04	5.82 ± 0.51	-13.82 ± 0.47
S269A	1.33 ± 0.32 x 10^6	0.80 ± 0.19	-7.91 ± 0.13	4.03 ± 0.70	-11.94 ± 0.57
S269D	NBD	---	---	---	---

(**a**) Binding of Su(H) protein to labelled DNA-oligo was competed with unlabelled DNA-oligo in the given concentrations; binding was assayed by electro-mobility shift as shown in Fig. 4a. Only Su(H)^{wt} and Su(H)^{S269A} bound well to be analysed. Signals were quantified by densitometry; no obvious differences in binding affinities were observed. DNA = TTGGGTGGCTCGTGG**CGTGGGAA**CCGAGCTGAAAG.

(**b**) Loading control of in vitro generated Su(H) protein isoforms used in the electro-mobility shift assay shown in Fig. 4a.

(**c**) Calorimetric binding data for Su(H) constructs binding to DNA. ITC experiments were performed at 10°C as shown in Fig. 4b. Values are the mean of two independent experiments, and the errors represent the standard deviation of multiple experiments. DNA = GTTAC**TGTGGGAA**AGAAAG.

Supplementary Figure S3 Effect of phospho-mutant Su(H) variants on Cut expression

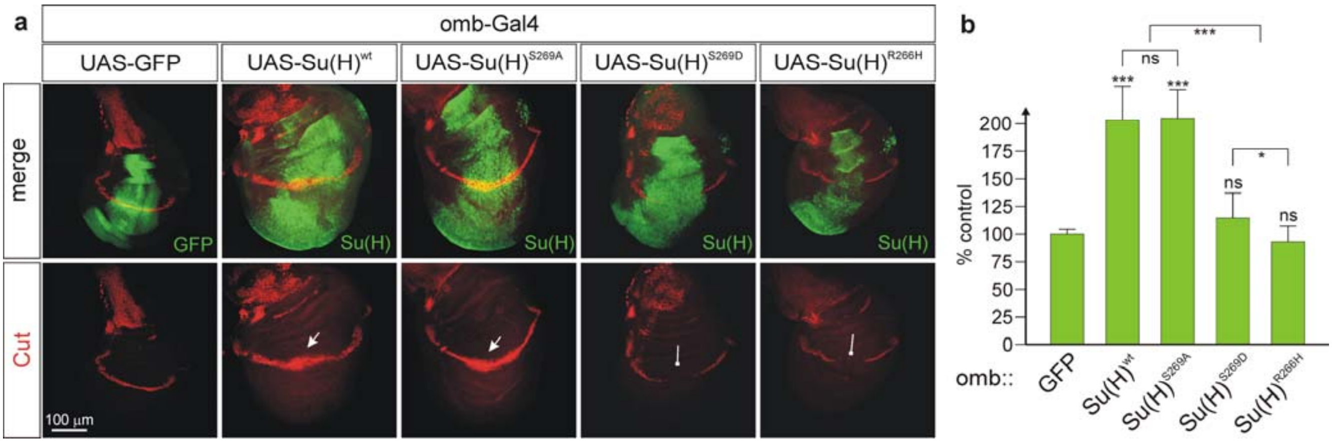

(**a**) Su(H) variants (green) as indicated were induced in the central part of the wing anlagen with the *omb*-Gal4 driver and expression of Cut (red) was analysed. UAS-GFP (green) served as control. Arrows point to enhancement of Cut expression; repressive beam to repression. Size bar represents 100 µm in all panels.

(**b**) Size of the area expressing the given Su(H) variant was measured using *Image J*; the control UAS-GFP was taken as 100%. Standard deviation (n = 10) is shown; ANOVA two tailed Tukey/Kramer was used for statistical evaluation; *** p <0.001; ** p <0.01; * p <0.05; ns p > 0.05 (not significant). Symbols on top of the bars represent comparison to GFP control; other comparisons are as indicated by horizontal lines.

Supplementary Figure S4 Consequences of combined overexpression of Hairless and phospho-mutant Su(H) variants on Cut expression

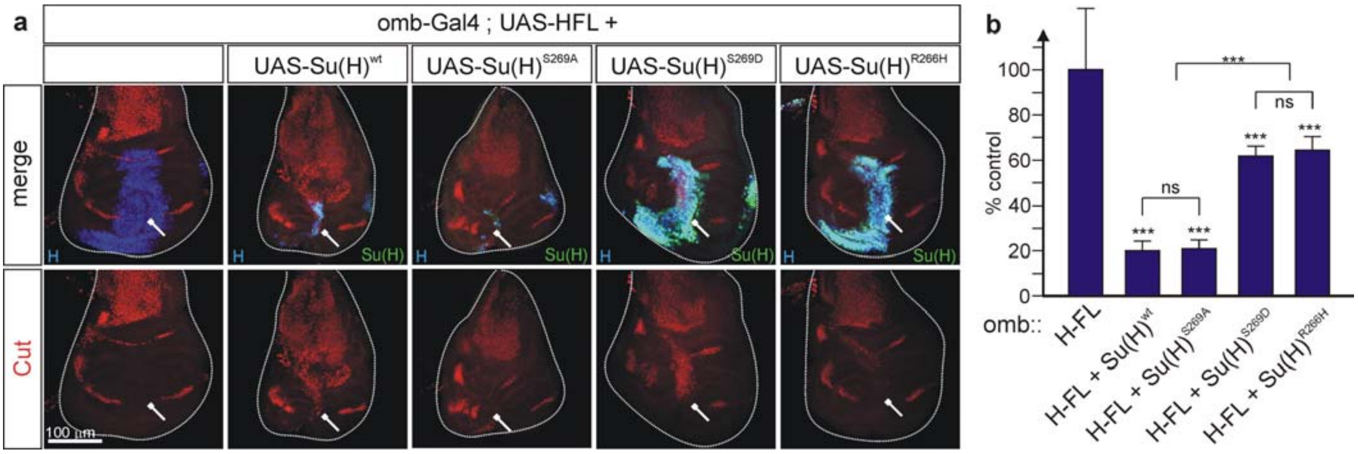

(**a**) Overexpression of UAS-H (blue) using *omb*-Gal4 represses Cut expression (red) at the d/v boundary (repressive beam). Co-overexpression of either UAS-Su(H)^{wt} or UAS-Su(H)^{S269A} (green; appears turquoise overlaying blue signals) together with UAS-H enhances repression in contrast to either UAS-Su(H)^{S269D} or UAS-Su(H)^{R266H}. Size bar represents 100 µm in all panels.

(**b**) Quantification of the area expressing the given Su(H) variant together with full length H (H-FL) was done using *Image J*; the control UAS-H was taken as 100%. Standard deviation (n = 10) is shown; ANOVA two tailed Tukey/Kramer was used for statistical evaluation; *** p <0.001; ** p <0.01; * p <0.05; ns p > 0.05 (not significant). Symbols on top of the bars represent comparison to H-FL control; other comparisons are as indicated by horizontal lines.
